# Comparison of Antiviral Activity between IgA and IgG Specific to Influenza Virus Hemagglutinin: Increased Potential of IgA for Heterosubtypic Immunity

**DOI:** 10.1371/journal.pone.0085582

**Published:** 2014-01-17

**Authors:** Mieko Muramatsu, Reiko Yoshida, Ayaka Yokoyama, Hiroko Miyamoto, Masahiro Kajihara, Junki Maruyama, Naganori Nao, Rashid Manzoor, Ayato Takada

**Affiliations:** 1 Division of Global Epidemiology, Research Center for Zoonosis Control, Hokkaido University, Sapporo, Japan; 2 School of Veterinary Medicine, the University of Zambia, Lusaka, Zambia; Public Health Agency of Canada, Canada

## Abstract

Both IgA and IgG antibodies are known to play important roles in protection against influenza virus infection. While IgG is the major isotype induced systemically, IgA is predominant in mucosal tissues, including the upper respiratory tract. Although IgA antibodies are believed to have unique advantages in mucosal immunity, information on direct comparisons of the in vitro antiviral activities of IgA and IgG antibodies recognizing the same epitope is limited. In this study, we demonstrate differences in antiviral activities between these isotypes using monoclonal IgA and IgG antibodies obtained from hybridomas of the same origin. Polymeric IgA-producing hybridoma cells were successfully subcloned from those originally producing monoclonal antibody S139/1, a hemaggulutinin (HA)-specific IgG that was generated against an influenza A virus strain of the H3 subtype but had cross-neutralizing activities against the H1, H2, H13, and H16 subtypes. These monoclonal S139/1 IgA and IgG antibodies were assumed to recognize the same epitope and thus used to compare their antiviral activities. We found that both S139/1 IgA and IgG antibodies strongly bound to the homologous H3 virus in an enzyme-linked immunosorbent assay, and there were no significant differences in their hemagglutination-inhibiting and neutralizing activities against the H3 virus. In contrast, S139/1 IgA showed remarkably higher cross-binding to and antiviral activities against H1, H2, and H13 viruses than S139/1 IgG. It was also noted that S139/1 IgA, but not IgG, drastically suppressed the extracellular release of the viruses from infected cells. Electron microscopy revealed that S139/1 IgA deposited newly produced viral particles on the cell surface, most likely by tethering the particles. These results suggest that anti-HA IgA has greater potential to prevent influenza A virus infection than IgG antibodies, likely due to increased avidity conferred by its multivalency, and that this advantage may be particularly important for heterosubtypic immunity.

## Introduction

It is known that both IgA and IgG antibodies play important roles in protection against influenza virus infection [1], [2]. While IgG is the major isotype of antibodies important for systemic immunity, IgA is predominantly present in mucosal tissues, including the upper respiratory tract, providing the first line of defense in mucosal immunity at the primary site of influenza virus infection. IgA antibodies are well documented to have unique properties in mucosa. Polymeric IgA (p-IgA) antibodies with a secretory component are selectively transported to the mucosal surface and resistant to proteolysis in mucosal secretions [2]–[7], and p-IgA antibodies crossing through epithelial cells via transcytosis are believed to inhibit viral protein functions intracellularly [2], [4], [6], [8]–[10]. In addition, p-IgA does not induce an inflammatory reaction in mucosa [2], [4], [7]. Therefore, it has been suggested that induction of the mucosal immune response is more desirable to prevent respiratory infection by influenza A viruses [11]–[14].

Influenza A viruses are divided into subtypes based on the antigenicity of two envelope glycoproteins, hemagglutinin (HA) and neuraminidase (NA). To date, H1-H16 HA and N1–N9 NA subtypes have been found in wild aquatic birds, the natural reservoir of influenza A viruses [15]–[17]. It is well documented that HA is the major target of neutralizing antibodies against influenza viruses. Since HA-specific IgG antibodies induced by subcutaneous or intramuscular injection with inactivated influenza vaccines are principally subtype-specific, protective effects are limited to viruses whose antigenicity is closely related to those of the vaccine strains [18], [19]. However, previous studies experimentally demonstrated that B-cell-dependent heterosubtypic immunity was induced by intranasal immunization of mice with formalin-inactivated viruses, whereas systemic immunization only protected mice from viruses with homologous HA subtypes [20]–[22]. We recently reported that both subcutaneous and intranasal immunization of mice with inactivated viruses induced antibodies that bound to HAs of multiple subtypes, but IgA antibodies showed greater ability than IgG antibodies to reduce plaque formation of viruses with heterologous subtypes [23], suggesting different antiviral potentials for IgA and IgG antibodies in heterosubtypic immunity.

We previously reported that an HA-specific monoclonal antibody (MAb) S139/1 (IgG) that originated from mice immunized with a virus of subtype H3 showed broad cross-reactivity to viruses with multiple HA subtypes, including H1, H2, H3, H13, and H16 [24], [25]. In this study, a cell line producing p-IgA was subcloned from an S139/1 IgG-producing hybridoma with spontaneous class switching in vitro. By using these monoclonal S139/1 IgA and IgG antibodies recognizing the same epitope, we compared their in vitro antiviral activities against influenza A viruses of the H1, H2, H3, and H13 HA subtypes.

## Materials and Methods

### Viruses and cells

Influenza A virus strains, A/Aichi/2/1968 (H3N2) (Aichi/H3), A/WSN/1933 (H1N1) (WSN/H1), A/Adachi/2/1957 (H2N2) (Adachi/H2), and A/gull/Maryland/704/1977 (H13N6) (Maryland/H13), were kindly provided by Dr. H. Kida, Graduate School of Veterinary Medicine, Hokkaido University, Sapporo, Japan. These viruses were propagated in the allantoic cavity of 10 day-old embryonated chicken eggs at 35°C for 48 hours. Virus particles were concentrated and purified by high-speed centrifugation of the allantoic fluid passed through a 10–50% sucrose density gradient. Purified viruses were disrupted with 50 mM Tris-HCl (pH 7.8) containing 0.5% Triton X-100 and 0.6 M KCl, and used as antigens for enzyme-linked immunosorbent assay (ELISA) [24], [26]. Madin-Darby canine kidney (MDCK) cells were maintained in Eagle's minimal essential medium (MEM) (GIBCO) supplemented with 10% calf serum. Hybridoma cells producing MAb S139/1 IgG, which were previously generated by immunization of BALB/c mice with formalin-inactivated purified Aichi/H3, were used [24]. Hybridoma cells were cultured in RPMI 1640 medium supplemented with 10% fetal bovine serum.

### Generation of hybridoma variants producing p-IgA

To obtain hybridoma variants producing p-IgA antibodies recognizing the same epitope as S139/1 IgG, we modified a sib selection/ELISA method that relied on spontaneous class-switching of the cells [27], [28]. Hybridoma cells were first grown in 96-well microplates at 1,000 cells per well and incubated at 37°C in the presence of 5% CO_2_. At 70% to 90% confluence, culture supernatants were screened for IgA production by sandwich ELISA using immunoplates previously coated with goat anti-mouse IgA (BETHYL). Cells from positive wells were harvested and replated at 10–100 cells per well. Screening for IgA and replating were repeated several times and IgA-producing cells were finally established from a single cell clone. Messenger RNA was extracted from the variant hybridoma clone using an RNeasy Mini Kit (QIAGEN). The variable region gene was amplified by reverse transcription and polymerase chain reaction using a One-Step PCR Kit (QIAGEN) with primers 5′-GAT GGT GGG ATT TCT CGC AGA CTC-3′ and 5′- SAR GTN MAG CTG SAG SAG TC-3′ for the heavy chain, and 5′- GGA TAC AGT TGG TGC AGC ATC-3′ and 5′- GAY ATT GTG MTS ACM CAR WCT MCA-3′ for the light chain, followed by direct sequencing. We confirmed that the amino acid sequence of the variable region of S139/1-derived IgA was identical to that of the original S139/1 IgG [25]. S139/1 IgA and IgG interfered each other in a competitive antibody binding assay (Figure S1) and did not show hemagglutination-inhibition (HI) activities against WSN/H1 and Aichi/H3 escape mutants obtained previously [24], confirming the same epitope recognition of S139/1 IgA and IgG.

### Purification of MAbs

Hybridoma cells producing S139/1 IgA and IgG antibodies were cultured in BD Cell MAb Medium, Serum Free using CELLine Flask (BD). S139/1 IgA and IgG antibodies were purified from culture supernatants using KAPTIV-AE™ (TECNOGEN) and Protein G Sepharose 4 Fast Flow (GE Healthcare), respectively, according to the manufacturers' instructions. MAb concentrations were measured by optical density at 280 nm using a NanoDrop 1000 spectrophotometer (Thermo Fisher Scientific). Purified MAbs were analyzed by SDS-PAGE under reducing or nonreducing conditions. For reducing conditions, MAbs were mixed with SDS-PAGE sample buffer with 2-mercaptoethanol (2-ME) (Wako) and heated at 98°C for 5 minutes. For nonreducing conditions, MAbs were mixed with SDS-PAGE sample buffer without 2-ME and incubated at room temperature for 30 minutes. Protein bands stained with Quick-CBB PLUS (Wako) were quantified using a VersaDoc™ Imaging System (Bio-Rad) and Image Lab™ software (Bio-Rad).

### Binding assay

Binding of MAbs was measured by ELISA as described previously [29]. Briefly, ELISA plates (Nunc Maxisorp) were coated with the disrupted influenza A virus antigens (Aichi/H3, WSN/H1, Adachi/H2, and Maryland/H13), and washed with PBS containing 0.05% Tween 20 (PBST), followed by blocking with 3% skim milk in PBS. Fourfold serially diluted MAbs in PBST containing 1% skim milk were plated in duplicate, and bound MAbs were detected using goat anti-mouse IgA (α) and goat anti-mouse IgG (γ) antibodies conjugated to horseradish peroxidase (Kirkegaard & Perry Laboratories, Inc.) diluted in PBST containing 1% skim milk. The reaction was visualized by adding 3,3′,5,5′-tetramethylbenzidine (TMB, Sigma-Aldrich) and the absorbance at 450 nm was measured. MAb concentrations required to give half-maximal binding (50% effective concentration: EC_50_) to influenza A virus antigens were determined using SoftMax® Pro 6.2.1 software (Molecular Devices) [30], [31].

### HI and neutralization tests

HI activities of the purified MAbs were tested by the standard method using 0.5% chicken erythrocytes. Neutralizing activities of MAbs were evaluated by the standard procedure of plaque-reduction tests using MDCK cells. Fourfold serial dilutions of MAbs (50 µl) were mixed with an equal volume of diluted virus solution (approximately 50–100 plaque-forming units), and incubated for 1 hour at room temperature. Then the mixture was inoculated onto a monolayer of MDCK cells on 12-well tissue culture plates. After 1-hour incubation at 35°C, the inoculum was aspirated and cells were washed once with serum-free MEM and overlaid with MEM containing 1% Bacto-agar and trypsin (5 µg/ml) (GIBCO). Plaques were counted after incubation at 35°C for 1 day (WSN/H1 and Adachi/H2), 2 days (Aichi/H3), or 3 days (Maryland/H13).

### Viral release inhibition assay

MDCK cells on 24-well plates were first inoculated with viruses at a multiplicity of infection of 1-2, followed by incubation for 1 hour at 35°C. The inoculum was aspirated, and the cells were washed three times and cultured with MEM containing S139/1 IgG or IgA antibodies (0.1 or 1 µg/ml) for 12 hours. Culture supernatants were collected at 0, 6, and 12 hours after infection, and mixed with SDS-PAGE sample buffer with 2-ME and treated at 98°C for 5 minutes. After 10% SDS-PAGE, separated proteins were blotted on a polyvinylidene difluoride membrane (Millipore). Chicken antiserum to Aichi/H3 was used as the primary antibody to detect viral proteins. The bound antibody was detected with peroxidase-conjugated rabbit anti-chicken IgY (IgG) (H+L) (Jackson Immuno Research, USA), followed by visualization with Immobilon Western (Millipore). Band intensities of the M1 protein were analyzed with a VersaDoc™ Imaging System (Bio-Rad) and Image Lab™ software (Bio-Rad).

### Electron microscopy

Transmission electron microscopy (TEM) was carried out as described previously [32]. For ultrathin sections, MDCK cells infected with viruses at a multiplicity of infection of 1-2 were cultured with MEM containing S139/1 IgA or IgG antibodies (1 µg/ml) for 8 or 12 hours and fixed for 20 minutes with 2.5% glutaraldehyde in 0.1 M cacodylate buffer (pH 7.4). The cells were scraped off the plate, pelleted by centrifugation, and then fixed for 30 minutes with the same fixative. Small pieces of the fixed pellet were washed with cacodylate buffer, postfixed with 2% osmium tetroxide in the cacodylate buffer for 1 hour at 4°C, dehydrated with a series of ethanol gradients followed by propylene oxide, embedded in Epon 812 Resin mixture (TAAB Laboratories Equipment Ltd), and polymerized at 60°C for 2 days. Ultrathin sections (70 nm) were stained with uranyl acetate and lead citrate and examined with a Hitachi H-7650 electron microscope at 80 kV.

## Results

### Polymeric structure of S139/1 IgA

**Figure 1:**
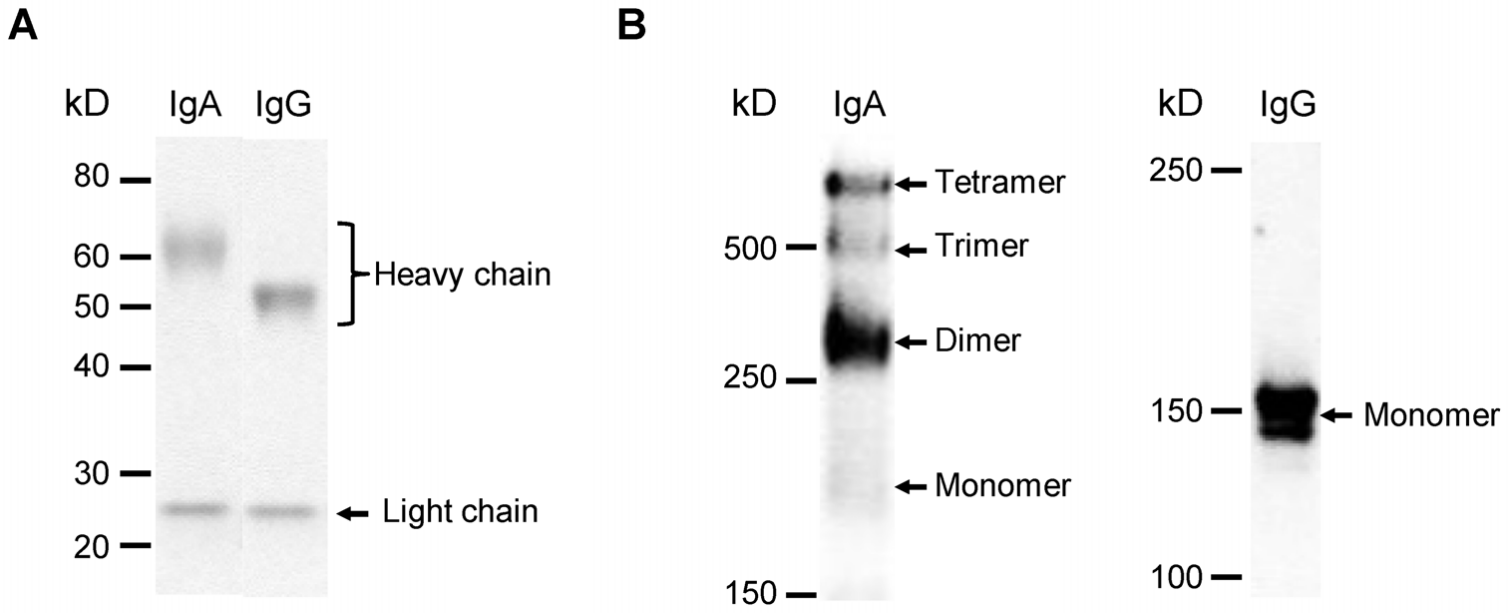
SDS-PAGE analysis of purified S139/1 IgA and IgG antibodies. Equal amounts (2.5 µg) of purified S139/1 IgA and IgG antibodies were analyzed by SDS-PAGE under reducing conditions (5%–20% gradient gel) (A). Polymeric forms of purified S139/1 IgA and IgG antibodies (5 µg) were analyzed under nonreducing conditions (3%–10% gradient gel) (B).

We first analyzed the purity and the molecular weight of purified S139/1 IgA and IgG antibodies by SDS-PAGE (Figure 1). Two protein bands corresponding to the light and heavy chains were exclusively visible in reducing conditions, thus confirming that the presence of impurities was negligible in the MAb preparations. In nonreducing conditions, while monomeric IgG antibodies of approximately 150 kilodaltons (kD) [4] were predominant, only a small amount of the monomeric form of IgA, which was expected to be an approximately 170-kD protein [33], was visible. Instead, three bands with molecular weights corresponding to dimeric, trimeric, and tetrameric IgA antibodies, were observed. Accordingly, these bands were also detected by immunoblotting with anti-mouse IgA (data not shown). Based on the quantified band intensities, we estimated that the relative amounts of monomeric, dimeric, trimeric, and tetrameric forms of IgA were approximately 1, 5, 1, and 3, respectively. These data indicated that the majority of S139/1 IgA produced from the hybridoma cells considered of p-IgA antibodies.

### Binding activities of S139/1 IgA and IgG antibodies to HAs of multiple subtypes

**Figure 2:**
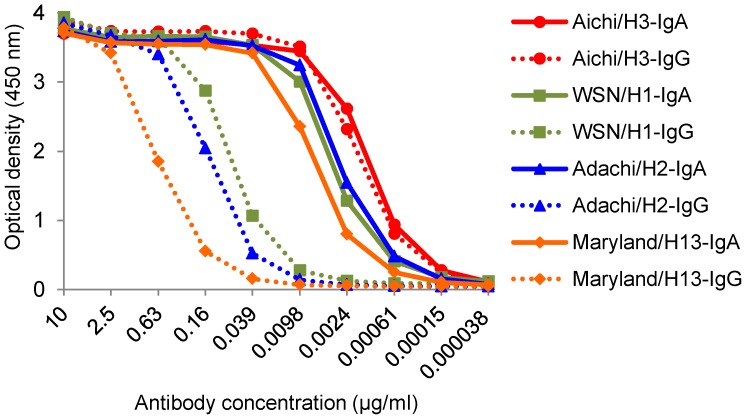
Comparison of binding activities of S139/1 IgA and IgG antibodies. Binding activities of S139/1 IgA (continuous lines) and IgG (dashed lines) were tested in ELISA. Disrupted viral particles of Aichi/H3, WSN/H1, Adachi/H2, and Maryland/H13 were used as antigens. Data are mean values of duplicate wells. EC_50_ values calculated based on the ELISA data are shown in Table 1.

We then investigated the binding activities of S139/1 IgA and IgG antibodies to the homologous HA antigen (Aichi/H3, which was used for production of this MAb) and heterologous HA antigens (WSN/H1, Adachi/H2, and Maryland/H13) by ELISA (Figure 2), and determined the EC_50_ values (Table 1). EC_50_ is generally used as a reasonable approximation of the dissociation constant (Kd) [30], [31]. We found that both S139/1 IgA and IgG antibodies showed similar binding curves for the Aichi/H3 antigen and that there was no significant difference in their EC_50_ values (0.0013 µg/ml and 0.0017 µg/ml, respectively). In contrast, there were remarkable differences between S139/1 IgA and IgG antibodies in reactivities to the WSN/H1, Adachi/H2, and Maryland/H13 antigens. IgA bound to these heterologous antigens at an extent similar to that with the homologous H3 antigen, and the EC_50_ values for WSN/H1, Adachi/H2, and Maryland/H13 were only 2- to 5-fold lower than that for the Aichi/H3 antigen. However, IgG reactivities to the heterologous HAs were uniformly much lower than that to the H3 antigen as indicated by the considerably larger EC_50_ values for WSN/H1, Adachi/H2, and Maryland/H13 (0.0777, 0.144, and 0.670 µg/ml, respectively) than for Aichi/H3 (0.0017 µg/ml).

**Table 1:** Comparison of binding abilities of S139/1 IgA and IgG to influenza A viruses.

	EC_50_ (µg/ml)*		
Virus	IgA	IgG	IgG/IgA
Aichi/H3	0.0013	0.0017	1.3
WSN/H1	0.0039	0.0777	19.9
Adachi/H2	0.0029	0.144	49.7
Maryland/H13	0.0063	0.670	106.4

The concentrations of MAbs giving 50% of maximal binding to influenza A viruses were determined according to regression curves obtained from ELISA. The assays were performed three times and the representative data are shown.

### HI activities of S139/1 IgA and IgG antibodies against influenza A viruses of different HA subtypes

We next compared HI activities of S139/1 IgA and IgG antibodies (Table 2). Consistent with their binding activities shown in ELISA (Figure 2), both S139/1 IgA and IgG antibodies exhibited the highest HI activity to Aichi/H3 and their endpoint concentrations were only slightly different. In contrast, although IgA showed lower HI activities to WSN/H1, Adachi/H2, and Maryland/H13 than to Aichi/H3, endpoint concentrations of IgA were much lower than those of IgG. As clearly indicated by the IgG/IgA ratios of endpoint concentrations, IgA showed significantly higher HI activity against heterologous viruses than IgG.

**Table 2:** Comparison of HI activities of S139/1 IgA and IgG antibodies.

	HI endpoint (µg/ml)*		
Virus	IgA	IgG	IgG/IgA
Aichi/H3	0.0244	0.0488	2
WSN/H1	0.0488	0.1953	4
Adachi/H2	0.1953	3.1250	16
Maryland/H13	0.1953	6.2500	32

The lowest MAb concentration that completely inhibited hemagglutination of each virus is shown. The assays were performed three times and the representative data are shown.

### Neutralizing activities of S139/1 IgA and IgG antibodies against influenza A viruses of different HA subtypes

**Figure 3:**
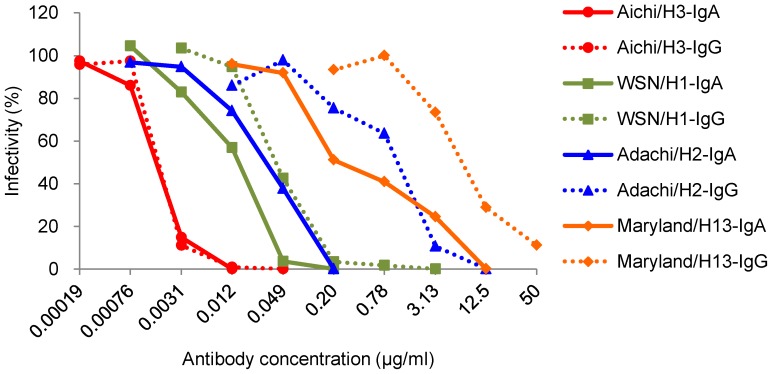
Comparison of neutralizing activities of S139/1 IgA and IgG antibodies. Appropriately diluted viruses were mixed with S139/1 IgA (continuous lines) or IgG (dashed lines) at the indicated dilutions. Neutralizing activities were evaluated by counting the number of plaques formed on MDCK cells. IC_50_ values calculated based on the neutralization curves are shown in Table 3.

We further compared the neutralizing activities of S139/1 IgA and IgG antibodies by the standard plaque reduction test (Figure 3) and the 50% inhibitory concentrations (IC_50_) of S139/1 IgA and IgG were determined (Table 3). Consistent with the similar binding and HI activities to Aichi/H3 (Tables 1–2 and Figure 2), both S139/1 IgA and IgG antibodies showed similar neutralization curves for this H3 virus and their IC_50_ values were almost indistinguishable. As was the case with HI activities, IgA showed remarkably higher heterosubtypic neutralizing activities to WSN/H1, Adachi/H2, and Maryland/H13 than IgG, as indicated by 4- to 23-fold differences of IC_50_ values (i.e., IgG/IgA), although both S139/1 IgA and IgG antibodies exhibited less neutralizing activity for heterologous viruses than for Aichi/H3.

**Table 3:** Comparison of neutralizing activities of S139/1 IgA and IgG antibodies.

	IC_50_ (µg/ml)*		
Virus	IgA	IgG	IgG/IgA
Aichi/H3	0.0015	0.0016	1.1
WSN/H1	0.0113	0.0445	3.9
Adachi/H2	0.0305	0.7117	23.3
Maryland/H13	0.4461	6.1585	13.8

IC_50_ values were calculated according to the neutralization curves shown in Figure 3. The assays were performed three times and the representative data are shown.

### Different potentials of S139/1 IgA and IgG antibodies to inhibit viral release from infected cells

**Figure 4:**
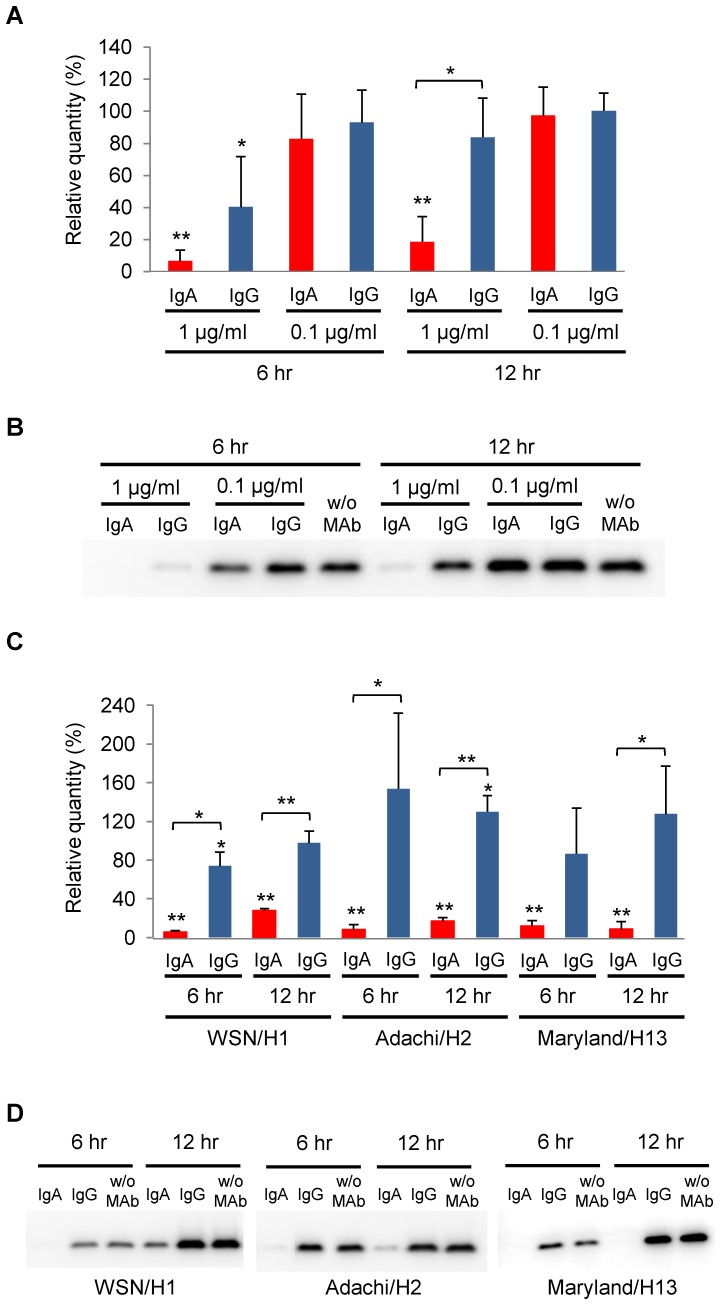
Viral release from infected cells cultured in the presence of S139/1 IgA or IgG antibodies. After inoculation with Aichi/H3 (A and B), WSN/H1, Adachi/H2, or Maryland/H13 (C and D), MDCK cells were cultured in the presence of S139/1 IgA or IgG antibodies at the concentrations of 1.0 or 0.1 µg/ml (A and B) and 1.0 µg/ml (C and D). Supernatants were collected 6 and 12 hours after infection, and viral proteins of influenza viruses released into the supernatants were detected by Western blotting (B and D). The relative quantity of the M1 protein was calculated based on the band intensity by using Image Lab version 3.0 (Bio-Rad) (A and C). The intensity of the M1 protein bands detected in the control supernatants collected from infected cells cultured without a MAb (w/o MAb) was set to 100%. Experiments were performed 3 times, and averages and standard deviations are shown (A and C). Statistical significance was analyzed by Student's t-test (**p<0.01, *p<0.05). Asterisks placed directly above the bars indicate significant differences compared to respective controls, and asterisks placed between the bars show significant differences between S139/1 IgA and IgG antibodies.

To compare the abilities of S139/1 IgA and IgG antibodies to suppress the extracellular release of virus particles from infected cells [23], MDCK cells infected with Aichi/H3, WSN/H1, Adachi/H2, or Maryland/H13 were subsequently cultured in the presence of S139/1 IgA or IgG antibodies, and the amounts of virus particles released into the culture supernatants were estimated by detecting the viral matrix (M1) protein in Western blotting (Figure 4A–D). We detected significantly lower amounts of the M1 protein of Aichi/H3 in the supernatants of infected cells cultured with S139/1 IgA (1 µg/ml) at both 6 and 12 hours after infection, whereas IgG had a smaller inhibitory effect only at 6 hours after infection (Figure 4A). At a lower concentration (0.1 µg/ml) of S139/1 IgA and IgG antibodies, no significant reduction was observed. Next, we performed the same assay for WSN/H1, Adachi/H2, and Maryland/H13 at the MAb concentration of 1 µg/ml and found that S139/1 IgA significantly suppressed the extracellular release of all tested viruses both at 6 and 12 hours after infection (Figure 4C and D). On the other hand, IgG showed limited activity only against WSN/H1 at 6 hours after infection. These data suggested that S139/1 IgA had greater potential to inhibit viral release from infected cells than IgG, which might be particularly important for the heterosubtypic antiviral potential.

### Aggregation of unreleased virus particles on the surface of infected cells cultured in the presence of S139/1 IgA

**Figure 5:**
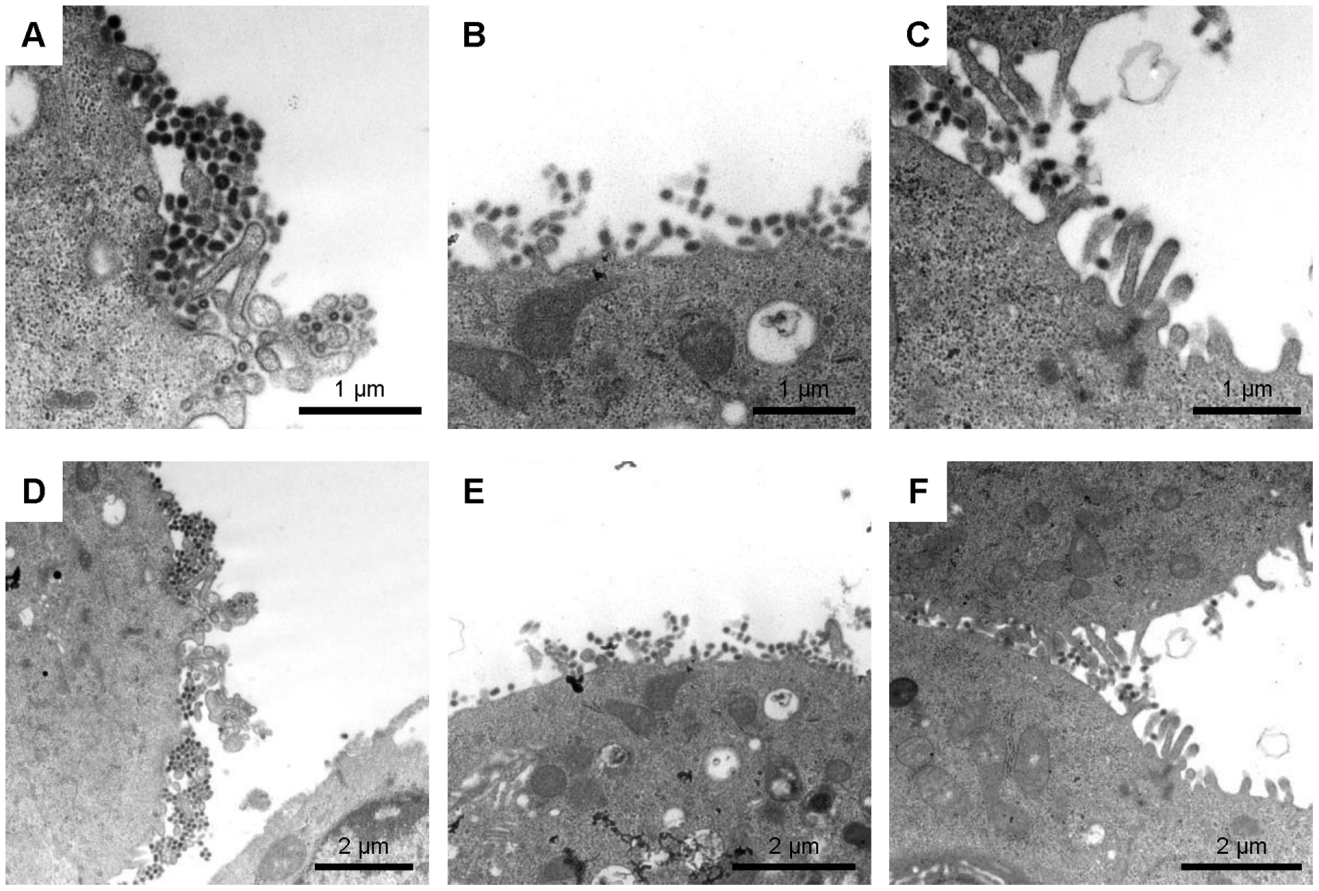
Viral particles deposited on the surface of infected cells cultured with S139/1 IgA. MDCK cells were infected with Aichi/H3 and incubated for 8 hours in the presence of S139/1 IgA (A and D), IgG (B and E), or in the absence of antibodies (C and F). TEM images of the cell surface are shown at high (A to C) and low (D to F) magnifications. Scale bars represent 1 µm (A to C) and 2 µm (D to F).

To gain insight into the mechanism of IgA-mediated inhibitory effects on the viral release from infected cells, we used electron microscopy to examine Aichi/H3-infected MDCK cells cultured with S139/1 IgA or IgG. After 8-hour incubation, tight aggregation and abnormal accumulation of unreleased virus particles were found on the virus-infected cells cultured in the presence of S139/1 IgA (Figure 5A and D), which was quite similar to the well-known phenomenon for the effect of dysfunction of the NA activity [34]–[37]. On the other hand, lower numbers of virus particles in less proximity were found on the infected cells cultured with IgG (Figure 5B and E). Only limited numbers of virus particles were seen on the surfaces of cells incubated without MAbs, suggesting efficient virus release from infected cells (Figure 5C and F). These data indicated that S139/1 IgA deposited newly produced viral particles on the cell surface more efficiently than S139/1 IgG. Interestingly, we observed that heavily accumulated and aggregated virus particles were likely incorporated into cytoplasmic vesicles in the cells incubated with S139/1 IgA at 12 hours after infection (Figure S2A–D). A similar observation was reported for MDCK cells infected with an NA-deficient influenza A virus [35]. The accumulation of densely aggregated virus particles in the cellular vesicles was hardly seen in infected cells cultured with S139/1 IgG (data not shown).

## Discussion

It has been reported that intranasal immunization induces a more efficient cross-protective immune response against influenza virus than systemic immunization, and IgA antibodies are suggested to play a major role in antibody-mediated heterosubtypic immunity [11]–[13], [20], [21], [38]. On the other hand, the currently used inactivated influenza vaccines, which rely on the induction of serum IgG antibodies, are believed to be effective only against viruses whose HA antigenicities are closely related to those of the vaccine strains. In this study, we directly demonstrated distinct differences in the heterosubtypic antiviral activities of IgA and IgG antibodies by using cross-reactive S139/1 IgA and IgG antibodies that had originally been produced against Aichi/H3 but recognized the same epitope shared among multiple HA subtypes (e.g., H1, H2, H3, and H13) [24], [25].

In ELISA, while S139/1 IgG bound strongly to the homologous Aichi/H3 HA, its cross-binding activity to heterologous HAs (i.e., WSN/H1, Adachi/H2, and Maryland/H13) was appreciably lower, in accord with previous studies [24], [25]. Interestingly, however, the difference between the S139/1 IgA reactivity to Aichi/H3 and to the heterologous viruses tested was much less prominent. The different antiviral activities of S139/1 IgA and IgG antibodies were directly confirmed by standard HI and plaque reduction neutralizing tests. Consistent with the binding assay, S139/1 IgA had much higher HI and neutralizing activities against WSN/H1, Adachi/H2, and Maryland/H13 than S139/1 IgG, whereas S139/1 IgA and IgG antibodies showed similar activities against Aichi/H3 virus. These results may suggest the increased avidity conferred by the polymeric form of S139/1 IgA due to its multivalent binding. Since bivalent binding of the whole S139/1 IgG molecule was shown to be important for its avidity to HAs other than the H3 subtype [25], this might be an important feature for heterosubtypic reactivities of S139/1 IgA and IgG antibodies. Alternatively, it might also be possible that class-switching from IgG to IgA itself might enhance the affinity of monomeric antibody molecules (i.e., the Fab fragment) to a single epitope by altering the flexibility of constant heavy chain of IgA, as suggested by a previous study by others [39].

As evidenced by the well-known protective efficacy of influenza virus NA inhibitors, the viral budding/release process is a promising target for antiviral development [34], [36], [40]. We previously reported that some antibodies to viral surface proteins inhibited the budding or release of virus particles from infected cells in vitro [23], [41]. In particular, the extracellular release of influenza A viruses from infected cells was suppressed in the presence of cross-reactive IgA, but not IgG antibodies, induced by immunization of mice [23]. To directly confirm the differential abilities of IgA and IgG antibodies to inhibit influenza virus release from infected cells, we used anti-HA monoclonal IgA and IgG antibodies (i.e., S139/1 IgA and IgG) recognizing the same epitope in this study. We found that S139/1 IgA efficiently inhibited the release of progeny viruses of all tested subtypes (i.e., H1, H2, H3, and H13), but the effects of S139/1 IgG were significantly weaker than those of IgA and limited to the homologous subtype (i.e., H3) (Figure 4). It was also noted that, unlike HI and neutralizing activities against the H3 virus that were similar for S139/1 IgA and IgG antibodies, appreciable differences in the inhibition of viral release were observed between S139/1 IgA and IgG antibodies even against Aichi/H3. Electron microscopy also revealed a notable difference between S139/1 IgA and IgG antibodies in the ability to trap newly produced virus particles on the cell surface (Figure 5A, B, D and E, Figure S2). Taken together, these results suggest that the polymeric structure of IgA is particularly important for this inhibitory effect, since cross-linking and tethering activities are likely required for depositing virus particles on the cell surface [2], [41]–[43].

Based on this hypothesis, the epitope may also be an important factor, since efficient crosslinking is likely done by antibodies that target the globular head region of HA. Accordingly, S139/1 recognizes highly conserved residues in the receptor binding site of the HA molecule, and thus has strong neutralizing activity [24], [25]. However, we assume that the neutralizing activity that blocks viral entry into cells is not necessarily required for the inhibition of viral release from infected cells. Interestingly, our previous study suggested that HA-specific non-neutralizing IgA antibodies induced by intranasal immunization of mice might have such potential [23]. Conversely, it is also conceivable that not all neutralizing antibodies have cross-linking and tethering activities if their epitope locations do not fit the required conditions. Thus, the “classical” neutralizing activity is not the only indicator of a protective antibody that may play a role in heterosubtypic immunity against influenza A viruses.

The present study demonstrates that anti-HA S139/1 IgA has greater antiviral potential against influenza A virus infection in vitro than IgG, and the advantage of IgA is more prominent in heterosubtypic cross-reactivity. The polymeric structure might be important for the enhanced ability of IgA antibodies. In addition, our data suggest that tethering and depositing newly budded virus particles on the infected cell surface may generally be one of the antibody-mediated protective mechanisms against enveloped viruses and emphasize the importance of p-IgA in mucosal immunity.

## Supporting Information

Figure S1 **Competitive antibody binding assay using S139/1 IgA and IgG.** ELISA plates were coated with the disrupted virus antigens (Aichi/H3), followed by blocking with 3% skim milk in PBS. Tenfold serially diluted S139/1 IgG and IgA were plated as competitive antibodies, followed by incubation with S139/1 IgA and IgG (1 ng/ml), respectively. Bound IgA and IgG were detected using goat anti-mouse IgA (α) and goat anti-mouse IgG (γ) antibodies conjugated to horseradish peroxidase. The reaction was visualized by adding 3,3′,5,5′-tetramethylbenzidine and the absorbance at 450 nm was measured.
(TIFF)

Figure S2 **TEM images of Aichi/H3-infected MDCK cells cultured in the presence of MAb S139/1 IgA.** MDCK cells infected with Aichi/H3 at a multiplicity of infection of 1-2 were incubated for 12 hours in the presence of S139/1 IgA. Ultrathin sections were examined by TEM. TEM images are shown at low (A and B) and high (C and D) magnifications.
(TIFF)
