# Supplementary figures and images for: Comparison of Antiviral Activity between IgA and IgG Specific to Influenza Virus Hemagglutinin: Increased Potential of IgA for Heterosubtypic Immunity
Source: PLoS One. 2014 Jan 17;9(1):e85582. doi: 10.1371/journal.pone.0085582 (PMC3895000; doi:10.1371/journal.pone.0085582)

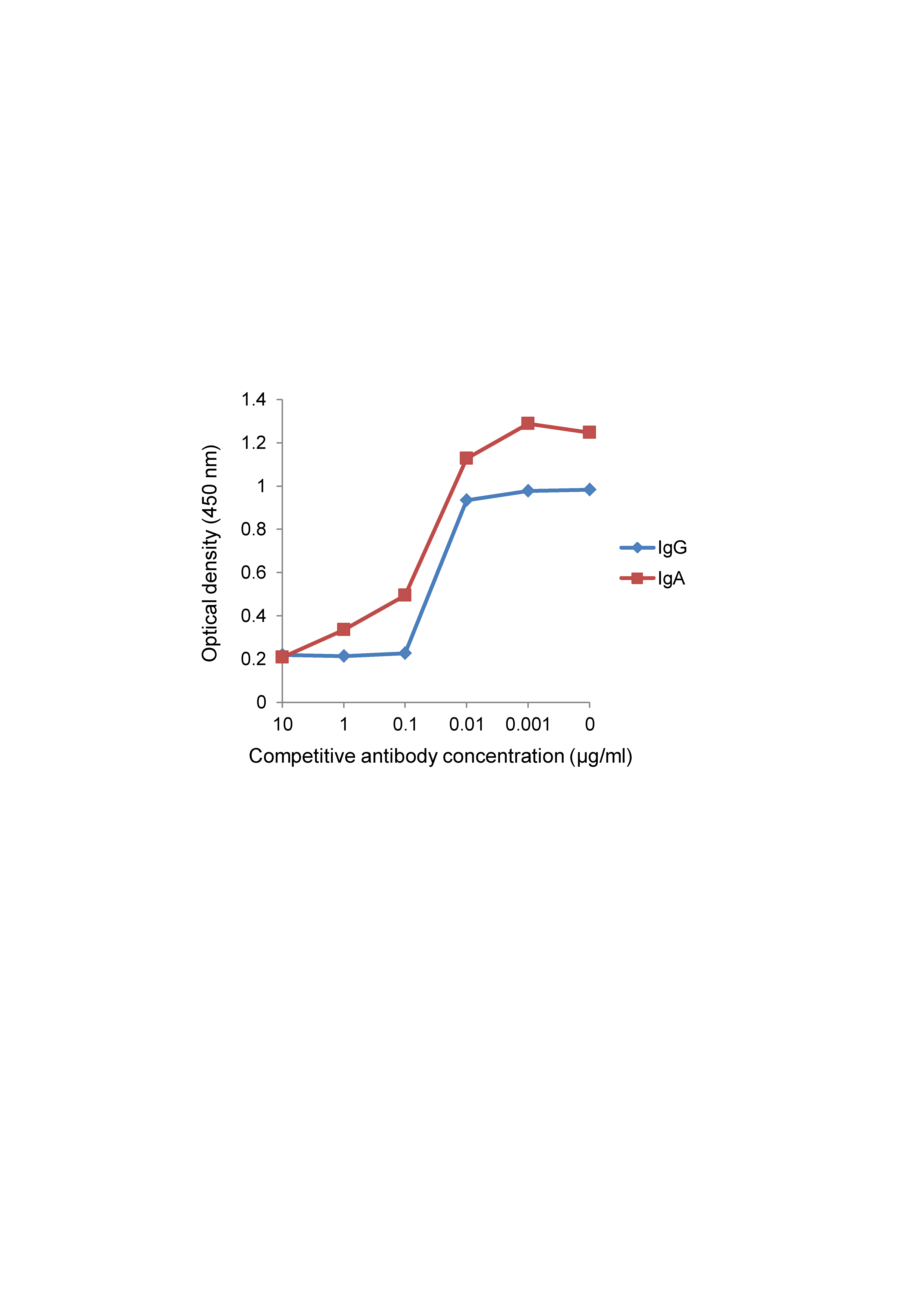

Supplement: Figure S1 — Competitive antibody binding assay using S139/1 IgA and IgG. ELISA plates were coated with the disrupted virus antigens (Aichi/H3), followed by blocking with 3% skim milk in PBS. Tenfold serially diluted S139/1 IgG and IgA were plated as competitive antibodies, followed by incubation with S139/1 IgA and IgG (1 ng/ml), respectively. Bound IgA and IgG were detected using goat anti-mouse IgA (α) and goat anti-mouse IgG (γ) antibodies conjugated to horseradish peroxidase. The reaction was visualized by adding 3,3′,5,5′-tetramethylbenzidine and the absorbance at 450 nm was measured. (TIFF) [file pone.0085582.s001.tiff]

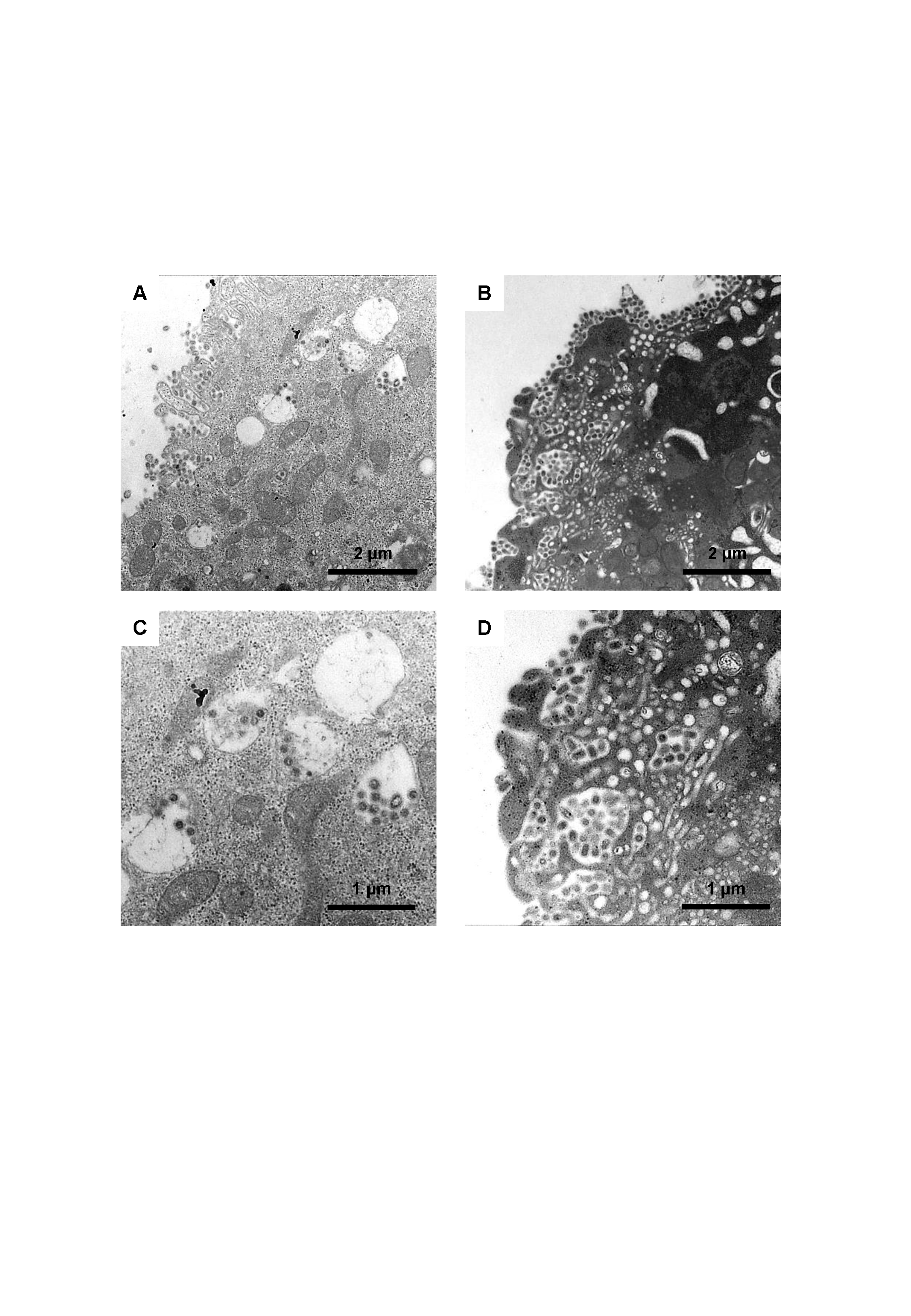

Supplement: Figure S2 — TEM images of Aichi/H3-infected MDCK cells cultured in the presence of MAb S139/1 IgA. MDCK cells infected with Aichi/H3 at a multiplicity of infection of 1-2 were incubated for 12 hours in the presence of S139/1 IgA. Ultrathin sections were examined by TEM. TEM images are shown at low (A and B) and high (C and D) magnifications. (TIFF) [file pone.0085582.s002.tiff]
